# Mental Health Challenges and Barriers to Veterans’ Adjustment to Civilian Life on the U.S.–Mexico Border

**DOI:** 10.3390/healthcare13030220

**Published:** 2025-01-22

**Authors:** Yok-Fong Paat, Angela V. Dorado, Nathan W. Myers, Andie Martinez, Shawna Scully

**Affiliations:** 1 Department of Social Work, The University of Texas at El Paso, El Paso, TX 79968, USA; 2 New Mexico State University, Las Cruces, NM 88003, USA; 3 Emergence Health Network, El Paso, TX 79901, USA

**Keywords:** mental health challenges, veterans, barriers to adjustment, U.S.–Mexico border

## Abstract

Background: Seeking mental health care is crucial for supporting effective reintegration among veterans. The U.S.–Mexico border presents a compelling and urgent case for study due to its proximity to economically marginalized and medically underserved areas, where the availability and accessibility of services are often limited. Objective: This study explored veterans’ mental health challenges and factors that hindered their adjustment to civilian life on the U.S.–Mexico border. Methods: A total of 36 veterans were recruited using purposive sampling from a mental health agency located in Southwestern Texas on the U.S.–Mexico border between November 2023 and May 2024 to complete an in-depth semi-structured interview and a brief survey. Results: Using thematic analysis, we found six themes associated with our study: (1) mental health struggles, (2) enduring military-influenced mindset, (3) adjustment to civilian life, (4) strained family relationships, (5) past victimization and discrimination, and (6) barriers to opportunities and mental health care. Conclusion: Understanding veterans’ mental health well-being and their prospects for integration into the civilian world is critical for identifying risk and protective factors that can inform the development of targeted health promotion initiatives, strengthen the implementation of equitable health care efforts, and support strategies for enhancing treatment access that address the unique needs of veterans in the border region. Policy and practice implications are discussed.

## 1. Introduction

For centuries, the United States service members have worked tirelessly to defend liberty, uphold justice, and safeguard the nation’s security [1]. In 2021, approximately 1.4 million Americans served on active duty in the military, while 800,000 were part of the reserve forces [2]. Although technological and medical advancements have increased the survival rates of service members in critical situations that would otherwise be fatal [3], many continue to be subjected to varied visible and invisible wounds from combat exposure, even following their military discharge [1]. In addition to facing elevated disease burden and health risks [4], veterans are more likely to report mental health risks and challenges than civilians [5]. Specifically, many military personnel and veterans report psychological concerns, including post-traumatic stress disorder (PTSD), depression, anxiety, alcohol and substance abuse, traumatic stressors from combat, and injuries (including disabilities) linked to cognitive impairment [6,7], which often lead to struggles with post-military life and challenges in reintegrating into civilian society.

Seeking mental health care is crucial to supporting successful reintegration. Yet, many veterans face considerable ecological barriers (e.g., stigma, awareness, service availability, challenges in navigating the healthcare system) to accessing mental health support vital for their well-being [8,9]. With the growing diversity of the U.S. veteran population, prioritizing health equity and understanding disparities in utilization, health care, and outcomes among ethnic/racial minorities and female veterans have become increasingly more critical than ever [10]. The U.S.–Mexico border presents a compelling and urgent case for study due to its rich cultural and linguistic diversity and its pressing need for mental health services. Proximity to the economically marginalized and medically underserved border can affect the availability and accessibility of services, as the region has historically suffered from a low provider-to-patient ratio, which can influence the types of care that veterans are able to access [11]. Ecological stressors and health disparities can negatively affect health and increase the risk of disease development [12]. Additionally, immigration rhetoric, political climate, geographical isolation, and border-specific stressors (e.g., immigration-related challenges, structural barriers), along with the predominately Hispanic demographic, may shape the provision of effective, community-tailored mental health services, veterans’ experiences with integration into civilian life, and access to social supports/networks [13,14,15].

While health disparities among veterans stemming from cumulative structural disadvantages have been documented in the current literature [16,17], the specific dynamics of racial/ethnic and gender disparities among veterans on the U.S.–Mexico border are not well understood. Understanding disparities in mental health and the utilization of mental health care among veterans on the U.S.–Mexico border is pivotal to ensuring equitable health care access, as mental health disorders can significantly contribute to our nation’s disease burden and healthcare costs [18], especially for a strategic location that plays a crucial role in shaping our nation’s cross-border health issues and enhancing health care access [19]. This study explored the mental health challenges and factors hindering veterans’ adjustment to civilian life on the U.S.–Mexico border, with a focus to shed light on the personal and systemic barriers they confront. Insights from this study can be used to inform the development of targeted health promotion initiatives, strengthen the implementation of equitable health care efforts, and support strategies for enhancing treatment access that address the unique needs of veterans in the border region.

## 2. Literature Review

### 2.1. Veteran and Military Mental Health

Military deployment can exert a far-reaching effect on the mental health of service members who experience a range of stressful events [7], with psychological symptoms typically peaking during the first year of deployment and may increase over time, even after departure from the military [20,21]. Empirical evidence suggests that veterans have poorer mental health than civilians, often manifesting higher rates of PTSD, depression, alcohol abuse, and comorbid conditions attributed to the physical demands and substantial stress incurred in life-threatening combat and military service [7,22,23]. There is evidence that suicide rates among U.S. active-duty soldiers and veterans have surpassed the rates among civilians [24]. Yet, many veterans with psychiatric disorders do not receive or utilize mental health services [25]. Veterans’ reluctance to seek care can worsen their psychosocial functioning and impede their quality of life and transition to civilian life [26]. In addition to encountering stress-related disorders, social difficulties, occupational difficulties [7], and service-related disabilities [27], veterans also report poorer health behaviors [4]. In particular, veterans are more likely to smoke, drink heavily, engage in substance use, exercise less, and have limited activities than civilians [28,29]. They also experience higher rates of obesity, cardiovascular disease [28], and traumatic brain injury than their civilian counterparts [30], along with complications associated with functional impairment [31,32].

Racial disparities among veterans, which can affect their mental health support and access to resources, have been observed across generations of military service [16]. Mental health-related discrimination, mistrust, and a lack of confidence in mental health services can lead to reduced engagement in treatment and care-seeking [33,34]. Veterans’ health disparities may stem from differential levels of stress exposure, childhood trauma, military roles, combat experience, health care access, and institutional barriers [35]. Even though timely access to health care is critical to optimal health outcomes, there is evidence that female veterans underutilize Veterans Health Administration (VA) health care relative to their male counterparts for many reasons, ranging from perceived affordability barriers and caregiver responsibilities to knowledge gaps in VA eligibility and services as well as perceived gender discrimination [36,37]. In particular, veterans with military sexual trauma experience an increased prevalence of mental health disorders [38]. The double disadvantage hypothesis noted that individuals with multiple disadvantaged identities face worse health outcomes than those with only one, highlighting compounded adverse effects exerted by intersecting disadvantages [39]. Women from racial and ethnic minority backgrounds, for instance, are more likely to face racial and gender discrimination concurrently [40]. They also report a higher rate of child abuse and interpersonal violence [41] and lower mental health treatment retention [42].

### 2.2. Reintegration into Civilian Life

Reintegration challenges can be shaped by multifaceted behavioral, physical, and social factors [7]. Combat deployment and military stressors (e.g., family separation, military-civilian cultural clash, and alienation from friends and families) can take a psychological toll and significantly impact the service members and their social circles (family members, social networks, and community) [43,44]. Indeed, deployment is associated with increases in parental stress, child behavioral challenges, and maltreatment [45]. Apart from relational disruption and turbulence during deployment, veterans may become less aware of their families’ challenges during their absence. Families may experience marital discord and challenges raising children, calling for the need to renegotiate roles and responsibilities [46]. On the one hand, service members’ dissatisfaction with their relationship can lead to depressive symptoms [47]. On the other hand, psychiatric symptoms (e.g., PTSD) may interfere with relationship functioning [48]. Female veterans may also feel compelled to protect their family and friends from the aftermath of war, PTSD, and depressive symptoms [49].

Many veterans experience maladjustment and difficulties coping with the transition into civilian life [50]. Many also struggle with post-combat externalizing behaviors (e.g., tobacco and substance use) [7] and experience reverse cultural shock [51]. In the social realm, a significant number of veterans experience feelings of social isolation and loneliness from civilian society, especially older veterans [52]. Even if reintegration, reunion with family, and return to civilian life are looked forward to, veterans face challenges and stressors, especially if the post-deployment transition is interrupted by traumas and disabilities. They must also successfully rebuild their competence to integrate [53]. While public stereotypes and stigma can pose significant deterrents to mental health service use and barriers to reintegration, hindering help-seeking [7], veterans may experience self-stigma by internalizing negative beliefs about their mental illness, leading to shame and guilt [54]. Internalized stigma has been linked to poor adherence to treatment and the use of mental health services [55]. Stigma faced by racial/ethnic minority communities has also been noted to reduce the utilization of mental health services [56]. Empirical evidence suggests that the rate of utilization of mental health services varies considerably across racial and ethnic groups, with the Hispanic community accessing mental health care significantly less than the White population [57]. Low English proficiency, limited educational attainment, inadequate health insurance coverage, provider and healthcare system mistrust, and a lack of culturally competent services are among the commonly cited barriers that compromise Latinos’ access to care and quality of care [58,59].

## 3. Method

Using protocol approved by the University’s Institutional Review Board (IRB #: 2071316), a total of 36 participants were recruited using purposive sampling from a mental health agency located in Southwestern Texas on the U.S.–Mexico border to complete an in-depth semi-structured interview and a brief survey. This sample size was deemed adequate for capturing participants’ shared experiences through qualitative data collection, as it was justified by the reach of data saturation, where further data collection yielded no new significant insights [60]. To be eligible for participation in the study, potential participants had to be at least 18 years old, U.S. veterans, and received mental health treatment for a minimum of 3 months at the time of the data collection. The interviews, which took place between November 2023 and May 2024 and lasted about 36.5 minutes on average, were recorded and transcribed verbatim. During the interview, the participants, who provided informed consent to participate in the study, were asked questions related to their mental health, social relationships, employability, education, support and assistance, personal histories, military service, psychosocial adjustment, barriers to services, satisfaction with treatment, therapeutic outcomes, adherence to their treatment plan, and outlook on life (see Supplementary Materials for interview questions). The interview questions were asked in a predefined sequence, allowing flexibility to delve into participants’ responses in greater depth when necessary and as additional insights came to light. Semi-structured interviews also enabled the research teams to ask follow-up questions for clarification, allowing participants to elaborate on their thoughts, actions, meanings, experiences, and insights through their voices [61]. To ensure the confidentiality of the participants, data were securely stored in password-protected files and portable flash drives while identifiable information was anonymized, ensuring that the participants’ privacy was safeguarded throughout the study.

We conducted thematic analyses to systematically describe the data and derive insights from the qualitative data [62,63]. First, the research team reviewed the transcripts line by line repeatedly to familiarize themselves with the data. Three research team members independently coded the data to minimize bias and increase the validity of the coding process. Collaborative team meetings were held to identify commonality, ensure consistency, compare and contrast diverse scenarios, highlight recurring or significant events, and identify patterns across the data. Initial codes were generated and organized into meaningful categories, which were then refined to develop coherent themes that capture key patterns and unique insights from the data that reflect participants’ perspectives and experiences. To increase the reliability of the coding scheme, discrepancies in coding were discussed and resolved through discussion, clarification, and consensus among research team members. Utilizing thematic analysis provides critical advantages in analyzing qualitative data as it systematically organizes and condenses large volumes of data, providing a nuanced understanding of the participants’ experience and perspective [64]. To safeguard the participants’ identity, only pseudonyms were used in this article. Given the small sample size, descriptive analyses were used to assess the survey responses, which helped us gain better insights into the attitudes, feelings, disposition, skills, and challenges of our participants (see Supplementals Materials for survey questions). We discussed the qualitative and quantitative findings concurrently below to provide a holistic understanding of our findings.

## 4. Participant Characteristics

About 58 percent of the participants were male. The average age of the participants was 42.4 years old. Regarding race and ethnicity, 41.7 percent were Hispanic/Latino, 25.0 percent were Black, 19.4 percent were Biracial, 11.1 percent were White, and 2.8 percent were Asian. About 44.4 percent of the participants claimed to be married, while three-quarters (75 percent) had children. With respect to nativity, nearly nine in ten participants (88.9 percent) were born in the U.S. About half (50 percent) were college graduates or claimed to be employed, respectively. In addition, approximately 22.2 percent were enrolled in an educational program, and an estimated 47.2 percent reported receiving financial assistance or having economic means to support themselves besides employment. Slightly over half of the participants (51.4 percent) had used other community services to cope with mental health. The majority of the participants (80 percent) acknowledged that they had health issues. Additionally, close to six in ten participants (63.9 percent) were taking medication as prescribed for their mental health. All aforementioned percentages were calculated based on available valid data.

## 5. Findings

**Figure 1:**
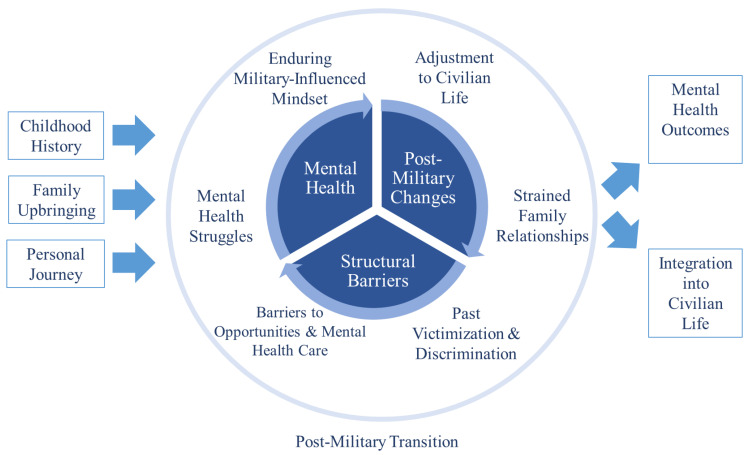
Mental health challenges and barriers to veterans’ adjustment to civilian life.

We found six themes associated with our study: (1) mental health struggles, (2) enduring military-influenced mindset, (3) adjustment to civilian life, (4) strained family relationships, (5) past victimization and discrimination, and (6) barriers to opportunities and mental health care. Table 1 shows the themes, sub-themes, illustrative texts, and direct quotes derived from the interviews. Figure 1 provides a graphic representation of the study findings.

### 5.1. Mental Health Struggles

In addition to experiencing general mental health issues that affect the broader population, participants also faced unique mental health challenges stemming from trauma related to their military service, such as PTSD and combat-related stress. Some of the symptoms were exacerbated by delays in seeking treatment or the persistent effects of untreated mental health conditions, which significantly increased the severity and magnitude of the complications. Despite the need for mental health services during his decade-long military career, Javier, one of the participants, had to postpone seeking treatment due to his fast-paced military life. He disclosed his need for treatment of his mood instability and had been receiving care in the last few months:

I suffer from … depression and … mild anxiety with insomnia, so … I could be jolly right now and then 30 minutes from now, five minutes from now, I think of something or… something triggers me, … my anxiety is just through the roof, and I start to panic. … I just lose sleep over it because it starts to eat me up, and I just don’t get any rest. And it just kind of builds up throughout the days … very quickly.

Many also sought treatment to effectively regulate overwhelming emotions, alleviate tension, manage social anxiety, cultivate meaningful connections, and reduce social isolation by gaining a deeper understanding of how past traumatic military experiences affected their emotional well-being and social lives. Recently retired from the military, Daniela, who sought treatment to cope with social anxiety, shared her ordeal and fear of leaving her house: “I can’t do [*sic*] big crowds. I can’t go to the store. … When I was stationed on deployment [*sic*], … I saw a lot of death, and [it] just kind of [*sic*] makes me extremely anxious and … have flashbacks and all that PTSD.” Some participants sought treatment to address feelings of betrayal and reconcile their challenges with trust. Gabriela, who was seeking treatment for her major depression and anxiety, shared her symptoms: “I have flashbacks and nightmares. I don’t … sleep well. … I’m on guard all the time. I don’t trust the system.”

Lack of motivation (e.g., frequently manifested as difficulty getting out of bed) was a significant concern for participants with depressive symptoms, often interfering with their ability to engage in daily activities, pursue goals, and maintain social relationships. Married with two teenagers, Alejandro shared his struggles to improve his mental health, fearing that it might jeopardize his current marriage, just as it did with his first marriage:

I’m constantly anxious, constantly looking after my shoulder. … Most days, I don’t wanna [*sic*] do anything, yet I do have the energy to do it. … Holidays like [the] 4th of July, I can’t go outside. It has impacted my life and my wife’s and my kids’ lives, so we don’t do a lot of activities outside anymore. … I’m trying to change, … you know, trying to be a better husband and dad.

Some participants disclosed experiencing chronic paranoia and a heightened state of vigilance and restlessness due to unresolved and prolonged exposure to combat-related stress, which interfered with their ability to discern perceived danger from actual danger. Estrella, who had difficulty running errands by herself due to her paranoia following her military sexual trauma, spoke of her difficulties:

It’s hard for me to hold a job … after separating from the military. … It’s just harder. … You are constantly … watching your back because of the paranoia that someone is coming for you. … You’re always wanting to be aware of your surroundings and where your possible exits are if something is to happen … like triggers, … backflashes… I have experienced in the military.

### 5.2. Enduring Military-Influenced Mindset

Military life also affected the participants’ mental health and general well-being even after they had left the service. Some continued to live with the trauma and consequences of military-induced health concerns, both psychological and physical. Alejandro shared his challenge: “You went from working 18 hours on a flight deck … to working eight hours. … I hardly get [*sic*] any sleep.” Long work hours aside, service members must also confront the mental strain brought upon by the unpredictable nature of the military lifestyle. Andrew, who struggled with the lingering effect of military trauma (including ducking to avoid the bomb explosion) and a recent divorce, recalled his experience:

A soldier’s mental health will decline from [*sic*] an average civilian, whether it be long work hours, a lot of missed family time, … rapid deployments. … You’re talking about [the] military. … That is war. … You don’t need “soft” war members, you need people ready to execute a certain way, so you’re raising a “machine” basically, and it will have an effect on you. … You’re gonna [*sic*] have to scream into that person’s ear, make sure they understand, or communicate it across loud noises.

Battling anxiety was a prevalent issue among the participants. Currently attending a peer support group in the hope of connecting with other veterans sharing similar experiences following her recent relocation and retirement from the military, Camila voiced her mental health challenges:

I felt like I was always under pressure, under stressful situations. … I think that has caused a lot of anxiety. And so now, because I went through so much anxiety, so many stressful situations, now, like any little thing that I go through … even though it’s something small,… my body and my brain interpret it as something big. … It impacts me emotionally and physically.

Military is a mission-oriented field where service members are guided to achieve mission-driven goals and commit to a higher cause [65]. It is also a future-oriented field that prioritizes foresight and ensures readiness to address potential threats, as related to Olivia, who recently retired. She spoke of her experience:

I always had to think in the future, and I always had to think, like steps, days, and years into the future, which, I mean, living in the future creates anxiety, and so I definitely blame my anxiety … on the military because you’re always running scenarios in your head for your job. Like … my jobs often … entailed running scenarios and constantly thinking forward and playing the “what-if game” all day long. And so, … when you’ve done it for so many years, it’s very hard to turn that off and just be present … and be present and accept the present and accept that you don’t have to be the person in control.

In the military, participants were also taught to be strong and tough, where the suppression of emotions was common. Omar shared how his service-related injuries contributed to his anxiety:

I was a leader there and am [*sic*] used to the culture there. … It doesn’t matter if you’re injured. It doesn’t matter if you’re sick. You never go to what is called [a] “sick call,” which is you never go to complain about it. You just … do it no matter what, or else you’re looked at as a bad leader. As a bad soldier, you’re looked at as weak. … You would get shamed.

Indeed, seeking mental health services was not a viable option for some who had been conditioned to suppress their pain and struggles to prioritize the military mission. This ingrained mindset of prioritizing duty over themselves led the participants to overlook their mental health needs while on duty, perpetuating a cycle where seeking mental health care was downplayed. Stacy, who was seeking treatment for her severe anxiety, also echoed Rachel’s point of view:

Being in the military, you’re kind of [*sic*] taught to … suppress everything that you have going on because the mission comes first. … So, we’re kind of [*sic*] taught to … bottle things up and not address things. And then, I think that’s kind of where all the mental health issues do come from, … the alcohol abuse, people getting into drugs and things because we’re suppressing so much [that] no one knows how to talk about things. And it doesn’t help really because if you have a soldier or if you are a major, … we’re on a mission, and they’re not mentally ok, it causes issues and then people have to pick up each other’s slack.

### 5.3. Adjustment to Civilian Life

The majority of the participants disclosed challenges in adjusting to and reintegrating into civilian life, as the public expectations of reintegration might not align with the complexities veterans must confront in reality. Some participants experienced personal confusion as they struggled to redefine their new post-military identity, as in the case of Olivia. She shared her sense of disconnection: “I couldn’t wrap my head around not being a soldier. … I had been a soldier for my entire adult life. … I just didn’t … fit in right with society.” Participants also shared the frustration of the public’s lack of understanding about veteran and military life, highlighting how misconceptions and stereotypes could add to their challenge in reintegration. Female participants, in particular, confronted the dilemma of having to establish credibility, overcome skepticism, gain recognition, and convince others of their contribution as a service member in a male-dominated field. Valeria highlighted the hurdles pertinent to the double standards females must face as veterans: “I think being a woman is hard. It feels almost like you have to try to convince people that … you did serve, and you did do certain things, so … it has created an issue for me in my transition.”

Some participants reported feeling confused, overwhelmed, and lacking direction in their post-military lives. Some participants found it arduous to let go of the structured military routine that they were accustomed to (e.g., waking up early in the morning), especially those who had served in the military for an extended period. Brian related: “The military tells you what to do, when to do it, how to do it. Here, on the outside world, it’s different. People are not gonna [*sic*] tell you that.” Even something as simple as finding a suitable wardrobe and being addressed by her first name were relatively new for Stacy. She shared another related challenge:

I’m used to … the non-stop and a lot of structure. … I learned [to] work smarter not harder. And I think civilian work life is like the complete opposite. Everyone is like [*sic*] super micromanaged. … It’s not very like [*sic*] team comradery. … I’m still adjusting there because I was a supervisor. … And now, I have a supervisor. … So, I’m still adjusting.

Likewise, Sean also longed for the structure the military provided:

When you’re in the military, you have your life planned out. … You knew your life [for] the next six months. … We knew our life. You get out, it’s just like, “What [are] you gonna [*sic*] do today? “I don’t know yet. Figure it out as you go. … It feels different. That was probably the worst adjustment I would say than anything not having my life schedule anymore. … I love [the] structure I had.

Few participants had a smooth transition into civilian life after departing from the military. Rather, many faced considerable obstacles. In order to reintegrate into civilian life, Antonia spoke of the importance of having support and mentorship in her transition:

When I first got out of the military, I was in a community college that had a really large veterans network. And I think they were kind of like the key to me being able to transition back out cause [*sic*] there were a lot of veterans who had been out for like a few years longer than I had. And so … they kind of [*sic*] helped guide me. … I guess learning etiquette again … like not being … loud and … argumentative. … The military is just really like aggressive, and so, they kind of [*sic*] helped ease me back in. … I think that’s the key to transitioning back. Having a support system … like a network of … veterans that [*sic*] know what you went through and … know what you need to go through in order to … transition back. … Otherwise, you’re like [*sic*] get stuck in that … military mentality.

### 5.4. Strained Family Relationships

Strained family relationships arising from communication difficulties and mismatched expectations were uncommon. A number of the participants reported being estranged from their family of origin. In contrast, others continued to maintain cordial ties but were cautious about sharing their personal challenges with their family, fearing that might cause unnecessary worries from their family because they felt like their family did not truly understand them. These tensions could lead to frustration, emotional disconnection, communication breakdown, and difficulty in seeking support from their family in their transition to civilian life. Caleb, who started experiencing symptoms of depression and anxiety since returning from Iraq, described how his military experience altered his relationship with his family: “Growing up, we were real [*sic*] close. And then when I went into the military and came back, I became more distant because of what I saw.” He continued: “One of my buddies got killed. His room was next to mine. … When a mortar … went off, it hit his room.” Some participants claimed their families did not understand what they went through in service. Rachel revealed how the military had altered her relationship with her family, noting that while she had moved forward, her family remained stagnate:

I struggle in communicating, and they struggle with … communicating back with me. … Twenty years of being in the military can change a person. And I noticed that my family don’t know how to communicate but the simple way is to come forward and ask. But they have a … picture mentality that I’m the same person they knew me … before the military.

Some participants preferred to isolate themselves from their families. Sofia disclosed how her mental health state had strained her relationship with her family, leading her to distance from them:

With the paranoia, … everything is feeling on edge. It’s kinda [*sic*] like a little domino effect. You kinda [*sic*] start to feel anxious. And after feeling anxious, you kind of [*sic*] get tired of feeling anxious. So then, there’s like this sense of irritability and anger that kind of [*sic*] follows. And it’s like a cyclical thing that just kind of [*sic*] happens all the time. … So when that happens … most of the time, I can feel myself pulled away from my family.

After many years of delay in seeking help following her sexual trauma in the military, Valeria noted how military experience can shape service members’ familial relationships in varied ways. She provided a detailed account of how her struggles had affected her parenting and her efforts to reconcile with her children:

I used to do a lot of asking them. … “Let me know what I can do better.” And we started with the communication, and it was a lot of … hard truths at first. But I feel like now that my daughter is 19 and my son is 17, … I’m glad that I had that self-realization that … “Let me have this conversation with you now, and if there’s something I can do better and, I wanna [*sic*] apologize. Let’s not carry it into adulthood because I’d be damned … if you’re 30, and you’re fucking telling me something about when you were 7. … Let’s go through this now.” … But not everybody communicates with their kids like that. I’ve got a lot of friends that [*sic*] get out, and I see that … trickled effect onto their kids. And then, I have friends that [*sic*] are military kids that [*sic*] are adults now. And to see the isolation and … just the way that they behave, … I’m just telling you: military kids should get looked at as well.

### 5.5. Past Victimization and Discrimination

About 69.4 percent of the participants reported having been victimized in the past, and slightly over half of the participants (55.6 percent) indicated having experienced stigma at some point. Approximately 38.9 percent revealed having encountered adverse experiences, particularly childhood maltreatment by a family member (e.g., sexual abuse, physical abuse, mental abuse, neglect). Hispanic participants, in particular, were more likely to report enduring harsh physical punishment from their parents. Past victimization and traumatic experiences (even if they are non-military related) could compound their mental health struggles, increase their susceptibility, and impair their ability to cope during and after their military careers. Jessica, who was receiving group therapy for her PTSD and suffered from low self-esteem, spoke of the repercussions of her childhood abuse that amplified her trauma from the military:

I’ve gone through therapy as an adult, and able to reflect and identify some of those barriers that you don’t always recognize that they’re suppressed. And then, you just kind of [*sic*] develop an armor, so to speak, and you get through military life and … then things happen in the military. And your flight-or-fight responses are heightened compared to maybe somebody else that [*sic*] didn’t have that same type of childhood that their response is different.

Racial discrimination posed a challenge for some former service members of color, as they encountered persistent bias, unequal treatment, limited opportunity for career advancement, and even a hostile work environment, all of which hindered their full participation and resulted in systemic mistreatment. Admitted to being hypervigilant and cautious around others, George spoke of the racial discrimination he endured: “When I was stationed in [name of place], … I was the only black marine in my unit. And … I was treated … pretty poorly. … The other marines in my unit called me “nigger,” “coon.” … They would come and flip me out of my rack at night when I was sleeping.” Similarly, Ryan shared the lack of guidance in the military caused by discreet discrimination, which prompted him to leave the military to find other employment opportunities: “My career ended short. … My entire goal was to retire [from the military], and it didn’t happen. … I didn’t have the right information.”

Discrimination and victimization were not restricted to race but also occurred based on gender, which went beyond mere differential treatment to encompass the emotional, psychological, and physical toll that left the participants feeling powerless and traumatized. Valeria, who had first-hand experience with military sexual trauma, related:

I think being in the military, being a woman, … there’s just like a lot of men that [*sic*] typically like “leadership men” that [*sic*] try and make you do things for their own pleasure. It may not be sexual, but it might just be like, “I wanna [*sic*] have control over making you jump when I say ‘jump.’ So jump, and if you don’t do it, you’re being insubordinate. I’m a sergeant. … How dare you!” … That happens a lot.

In the same vein, Isabelle talked about the reality of gender discrimination and conflicts surrounding gender norms in the military:

Being a woman in the military is still hard. … You’re looked [at] as the weaker sex. And the army, … they try to mold you into a leader, so … you encounter sexual harassment no matter what you look like. And not only that, … you have … the men that [*sic*] are “You’re weak, how can you lead me into war?”

Jessica, who received more adverse treatment, shared her victimization experience in the military: “I was raped while I was in the military … as part of a hazing situation. For a long time, I lived with that victim mentality. But as soon as I started getting mental health services, it changed my outlook.”

### 5.6. Barriers to Opportunities and Mental Health Care

Only half of the participants were employed at the time of the interview, and about 19.4 percent claimed to have trouble getting and keeping a job. When asked to describe mental health-related factors that had limited their life opportunities, some admitted to being uncomfortable around people to the extent that they lost jobs and life opportunities. Some sought isolation to find peace, avoiding signs of weakness and vulnerability. Some also highlighted the perceived differences in work ethic between military and civilian workplaces. Lastly, some were not able to work due to health issues (fatigue, sleep issues, pain) and mental health issues (anxiety, panic attacks). Even with a master’s degree, Olivia noted that her mental health condition hindered her from seeking employment: “Sometimes the anxiety gets so crippling that … I may forget what day of the week it is. … Sometimes, I’ll have panic attacks, and it’ll be so bad that I can’t leave the house.”

Lack of sleep, mood changes, and being hypervigilant were some obstacles that hindered some participants from moving forward. George, who was unemployed at the time of the interview, shared his challenge:

Being hypervigilant is very tiring. … I live in a world where I look in every crevice for danger. … I’m looking for my threats, and it doesn’t go away. … It’s very tiring. When I go into the store, I go in, I get what I need, and I leave. I don’t wander around. … There’s [*sic*] too many people around. There’s [*sic*] too many possibilities of danger. So, for me, that is very debilitating to a certain extent because I don’t have any peace. I don’t have any peace at all.

He explained his hesitance and lack of confidence in taking advantage of potential job prospects: “I’m not gonna [*sic*] take advantage of opportunities that you might because you feel safe in doing it where I’m not safe. I don’t feel safe, so I don’t take that opportunity.” Indeed, participants who encountered obstacles in securing employment also reported diminished confidence and self-esteem, with their pessimistic outlook hindering them from actively pursuing available opportunities. Building social capital is critical to career success; however, challenges in mental health could impede their ability to cultivate and sustain these connections. Christopher, who was able to maintain his employment as a security officer for over a year, shared his challenge:

On my days off, I just wanna kind of [*sic*] shut down and try to shell up, like just “bunker down” in my bed for the three days I have off. … It makes it hard for me to get the momentum to go out and do things with other people that’s [*sic*] not required. … It makes it hard … to establish friendships or keep friendships. I’ve lost a lot of friends from my time until now.

Procrastination resulting from mental health challenges was another barrier that participants such as Javier struggled to overcome, in part because he found the issues so overwhelming and insurmountable that he felt powerless to overcome them:

I have a lot of anxiety … that came from service as well as the marriage that I had while in service. … Having the time to deal with it at the moment you’re having, it may be easier to do and affect you less long-term than having a problem and having to put it on the back burner because you don’t have the time to deal with it right now. … Think of it like this. You don’t pay your light bill this month, and then you’re like, “It’s whatever. It’s just like 50 bucks. Whatever, I’ll pay it next month.” And then you can’t afford it next month. And now, you have 100 dollars. … You kinda [*sic*] keep putting it off. And then, those months start to pile up, and you get those letters in the mail saying “Hey, you owe 800 dollars.” That’s kind of what it feels like when you’re in the army or in the service, you kind of like [*sic*] put it off over time. And then, that problem becomes so much bigger. And now, … you don’t know what to do. … And now, you’re anxious. You’re stressed. You’re depressed.

In addition to anxiety, self-doubts, discomfort in seeking help, and fear were identified as other significant obstacles by the participants in their journey for recovery and reintegration. In exploring the barriers hindering them from utilizing mental health services and keeping their mental health treatment appointments, we found that the everyday challenges included work schedules, fatigue, depression, and a fear of discussing sensitive information. Through therapy, participants learned to set good boundaries with others, develop good coping skills, and control their triggers. They also developed a higher threshold for tolerance and were better at communicating and relating to others.

## 6. Discussion

The United States is home to approximately 16.5 million veterans, comprising 6.4 percent of U.S. adults [66]. Since 2001, nearly 3 million service members have served in varied war operations, with over half deployed more than once [67]. Veterans returning from combat often experience a multitude of pressing mental health concerns, including depression, anxiety, PTSD, suicidal ideation, substance use, and other physical injuries or illnesses [68]. The U.S. Government Accountability Office (2021) estimated that the number of veterans receiving mental health treatment from the Department of Veterans Affairs is close to double, and the budget has tripled from USD 2.4 to USD 8.9 billion between 2006 and 2019. Further, it is projected that the costs of outpatient mental health care will continue to increase by 32 percent in the next decade, making mental health care and treatment for veterans a top national priority [69]. This study explored mental health challenges that veterans faced and factors hindering veterans’ adjustment to civilian life on the U.S.–Mexico border. Overall, we found that participants in our study struggled with mental health issues that complicated their adjustment to civilian life and routines, while structural barriers such as experiences with maltreatment and limited access to resources and support could further hinder their successful transition. We hope that insights from this study will help raise public awareness about the mental health needs of veterans suffering from mental health issues in border cities and develop infrastructure and interprofessional team collaboration to improve service provision.

Consistent with empirical studies from other regions [6,7], the participants’ mental health challenges persist following their discharge from their military service, with depression, PTSD, and anxiety as the most common issues reported. The military-influenced mindset, which prioritizes mission, strength, and duty [65], could make adapting to the less rigid and unpredictable civilian life challenging. Empirical evidence suggests that many veterans experience value clashes between civilian society and military communities, leading to a sense of disconnectedness and alienation [7]. Additionally, many feel the public does not fully understand the challenges that service members and veterans must confront [70]. Military culture’s emphasis on strength and resilience can lead to veterans’ fear of being perceived as weak and incompetent if they develop a mental illness [1], a finding indicated in our study. There is evidence that veterans avoid seeking help for fear that their record of mental illness can negatively impact their careers [71].

Further, we found that many experienced significant life challenges before and after their military service, had a history of victimization, including early childhood maltreatment and discrimination, and struggled with adjusting to post-military civilian life, including rebuilding family relationships. Blosnich et al. (2014) noted that adverse childhood experiences, which are linked to many health concerns, are more prevalent among the military, particularly for those who may enlist to escape dysfunctional homes and adverse household environments [72]. Our study revealed that experiences with discrimination exacerbated mental health struggles by fostering feelings of shame and isolation, further complicating the recovery process. In addition to being associated with poorer mental health outcomes, empirical evidence suggests that discrimination and stigma can deter veterans from seeking help by creating a hostile environment [73]. Many veterans experience delays in seeking help [74]; others are concerned about stigmatizing labels such as “crazy” and living up to the military expectation of “suck it up” [8]. Additionally, even though there is a multitude of resources and treatment options available for veterans today [1], many do not access them, as shown in this study. Johnson et al. (2018) indicated that veterans prefer mental health treatment from other veterans due to shared understanding and experience [75]. Yet, many of our participants also faced VA-related barriers in seeking care (e.g., difficulties in securing an appointment, eligibility issues).

Health disparities are preventable differences in health outcomes between subgroups, often linked to systemic environmental and socio-economic disadvantages [76]. Research examining disparities notes that racial/ethnic minorities are more likely to have persistent mental disorders and less likely to access or utilize mental health care [77]. They also report less satisfaction, poorer outcomes, and are more likely to drop out of mental health services [78]. Racial/ethnic disparities in health have been found among female veterans, with female veterans reporting higher rates of mental health disorders compared with their civilian counterparts or White counterparts [41,79]. Indeed, some of the female participants in our study experienced military sexual trauma during their service, highlighting the importance of targeted support and intervention to effectively address their mental health needs. It is important to note that women face different experiences in the military than men, which can have different repercussions on their health. Specifically, men and women express their mental health symptoms differently, with women more likely to internalize their symptoms while men are more likely to externalize them [80,81]. Further, young female veterans may face competing priorities, including work, school, and family responsibilities for caring for family and children, which can present barriers to mental health treatment compared to older women who already have established family and community lives but are more likely to face struggles with reintegrating after military service [80].

## 7. Practice and Policy Implications

Our study underscores the importance of interventions aimed at reducing barriers to improve access to mental health care and address quality of life disparities, particularly among racial/ethnic minorities and female veterans. The findings of this study can be used to guide the design and implementation of targeted mental health interventions for veterans, focusing on challenges identified in this study. Our study shows that policies that create targeted job training and placement programs that address unemployment or underemployment among veterans may significantly enhance their mental well-being and improve their transition to civilian life. Veterans may need education, employment, health care, financial support, legal services, and vocational rehabilitation to improve social functioning and daily operations, a finding supported by our study. Because veterans in our study often delayed seeking mental health care due to the demands of their fast-paced military career and other personal challenges, it is imperative for mental health practitioners and policymakers to develop and implement targeted outreach initiatives and accessible mental health services (e.g., telehealth, mobile clinic) that encourage veterans to seek mental health care as early evidence-based mental health treatment has been shown to effectively prevent mental health disorders and promote better long-term mental health outcomes [82,83].

It is critical that mental health professionals recognize that veterans have to navigate two overlapping identities shaped by military discipline and civilian life, which necessitates a culturally attuned therapeutic approach to address potential conflicts [84]. Maguen et al. (2010) recommended that therapists act sensitively within the context of supportive therapeutic relationships when working with veterans [85]. Veterans who have been conditioned to perceive emotional vulnerability as weakness may benefit from reframing their psychological scars as signs of strength and resilience [84]. Veterans may harbor ethical and moral concerns that can fundamentally alter their previous beliefs and values. Moral injury, for example, entails guilt, shame, and anger from witnessing and participating in a war zone. It can result from veterans’ commission or omission, acts perpetrated by comrades, or witnessing violence or injustice [86]. Service members may be skeptical of others’ motivation for fear of being judged or misinterpreted [43], which can impact their relationship with others. Veterans may need encouragement to revisit experiences they are otherwise hesitant to discuss. Hence, learning about their core values is essential to starting a conversation and reducing negative interactions that may delay or prevent them from seeking treatment [43]. Normalizing mental health, particularly among veterans along the U.S.–Mexico border, which has a higher concentration of Hispanics, is vital for improving community health, as stigma is linked to reduced help-seeking in mental health care, poor management of depression symptoms, reluctance to disclose mental health issues with family and friends, and non-adherence to antidepressant medication regimens among Hispanics [87].

Since many veterans present more than one mental health symptom, as shown in our study, mental health providers must assess the presence of comorbidity throughout the planning and treatment processes [88]. Integrating mental health screening in the primary care setting is critical for improving the early detection of mental health disorders [89], especially when working with ethnic/racial minorities to reduce the stigma connected with seeking mental health services. Hispanics, for example, face a range of barriers that hinder their access to care, including a lack of communication with their healthcare providers, the cultural stigma associated with mental illness, greater propensities to associate psychiatric symptoms with somatic complaints, and a higher likelihood of receiving mental health treatment from general health providers than mental health specialists [90]. Providers can help increase awareness about the functional needs of veterans by offering public psychoeducation about mental health care to reduce stigma and negative beliefs about mental health [25]. They may also consider engaging families in mental health services and inquiring about their cultural history and identity. This approach may help veterans improve communication with their family and peer support groups, foster trust and confidence in mental health services, and support their recovery. Expanding the availability of VA health care facilities in underserved border areas may increase access to mental health services.

## 8. Limitations

Due to the use of non-probability sampling and the small sample size in our research study, the findings are not intended to be broadly generalized. Given that our participant pool came exclusively from one organization and the study relied on voluntary participants receiving mental health treatment services, potential recruitment and self-selection bias might limit the representation and fail to capture the full diversity of the broader target population. Like any qualitative study, this study aims to provide an in-depth account of the participants’ life experiences and unique perspectives, prioritizing data richness over broad generalizability. Due to the small sample size and challenges in quantifying qualitative data, this study was unable to fully examine the link between the intersectionality of the participants’ demographics and mental health outcomes. Future research may consider exploring the intersectionality of veterans’ demographics by utilizing larger, more generalizable data sets to gain a comprehensive picture of the diverse challenges and needs veterans face.

## 9. Conclusions

Understanding veterans’ mental health well-being and their prospects for integration into the civilian world is critical for identifying risk and protective factors that can inform targeted interventions and support strategies to reduce health disparities among veterans on the U.S.–Mexico border. We found that veterans’ integration into civilian life was often hindered by several factors, including ongoing mental health struggles and enduring military-influenced mindset that made it challenging to adapt to civilian expectations and routines. Strained family relationships and their experiences with victimization and discrimination could exacerbate feelings of alienation and isolation. Adding to these challenges were barriers to opportunities and mental health care, which limited their access to essential support and resources necessary for successful integration.

## Figures and Tables

**Table 1:** Themes, sub-themes, illustrative texts, and direct quotes from the interviews.

Themes	Sub-Themes	Illustrative Texts and Direct Quotes from the Interviews
Mental health struggles	General mental health issues, mental health challenges from trauma related to military service	▪ Stress, anxiety, depression, PTSD ▪ “I don’t like to be around people no more.” ▪ Social anxiety, paranoia, and hypervigilance ▪ Coping with distrust and betrayal ▪ “I’m having trouble sleeping…getting through things.” ▪ A heightened state of vigilance and restlessness ▪ Poor mental health impaired life functioning and ability to perform daily activities.
Enduring military-influenced mindset	Physical health issues, psychological effects	▪ The military and civilian worlds are different ▪ Physical health issues and mental health strain from combat ▪ Service members are taught to be tough and strong ▪ “Military is a war machine.” ▪ Self-sacrifice and suppression of emotional needs to focus on military mission ▪ Mental health care and support were downplayed.
Adjustment to civilian life	Cultural and social norms, routine disruptions	▪ Public expectations did not align with veterans’ reality ▪ A sense of feeling out of place and loss of identity ▪ The civilian world lacks structure, routine, and discipline ▪ Social interactions as civilians were different ▪ The military provided a sense of belonging and camaraderie ▪ Different expectations and cultures in the civilian workplace ▪ Learning new etiquette outside of the military
Strained family relationships	Communication challenges, parenting issues	▪ Lack of understanding among family members ▪ Family members did not truly understand them ▪ Staying cautious around family members ▪ Emotional distance and family tensions ▪ Divorce and family break-ups ▪ Communication difficulties and role strain in parenting
Past victimization and discrimination	Childhood maltreatment, racial/ethnic and gender discrimination	▪ “I grew up in a very…physically and mentally abusive household.” ▪ Racial discrimination, bias, unequal treatment, and hostility ▪ Military sexual trauma and sexual harassment ▪ Powerless and traumatized ▪ Traumatic experiences exacerbated mental health struggles.
Barriers to opportunities and mental health care	Employment prospects, obstacles to moving forward, barriers to seeking care	▪ Poor mental health conditions limited life opportunities ▪ Lack of sleep, mood changes, hypervigilance, and procrastination ▪ Lack of confidence and discomfort around others ▪ Difficulty in cultivating meaningful relationships ▪ “A lot of the things…are weighing me down.” ▪ Anxiety, self-doubts, discomfort in seeking help ▪ Difficulties in maintaining mental health care

## Data Availability

Data are not available for public dissemination in order to protect the privacy of the participants.

## Supplementary Materials

The following supporting information can be downloaded at https://www.mdpi.com/article/10.3390/healthcare13030220/s1, Interview Questions and Survey Questions.

## References

[1] Botero G.J., Rivera N.I., Calloway S.C., Ortiz P.L., Edwards E., Chae J., Geraci J.C. (2020). A lifeline in the dark: Breaking through the stigma of veteran mental health and treating America’s combat veterans. J. Clin. Psychol..

[2] Gracey S., Keller A., Montgomery G., Raczniak G., Schumacher B., Studer N. (2023). United States Military Deployments: CDC Yellow Book 2024. https://wwwnc.cdc.gov/travel/yellowbook/2024/work-and-other-reasons/us-military-deployments.

[3] Vergun D. (2020). Medical Improvements Saved Many Lives During World War II. https://www.defense.gov/News/Feature-Stories/story/Article/2115192/medical-improvements-saved-many-lives-during-world-war-ii/.

[4] Hoerster K.D., Lehavot K., Simpson T., McFall M., Reiber G., Nelson K.M. (2012). Health and health behavior differences: U.S. military, veteran, and civilian men. Am. J. Prev. Med..

[5] Moore M.J., Shawler E., Jordan C.H., Jackson C.A. (2023). Veteran and Military Mental Health Issues. https://www.ncbi.nlm.nih.gov/books/NBK572092/.

[6] Dworkin E.R., Bergman H.E., Walton T.O., Walker D.D., Kaysen D.L. (2018). Co-occurring post-traumatic stress disorder and alcohol use disorder in U.S. military and veteran populations. Alcohol. Res..

[7] Elnitsky C.A., Blevins C.L., Fisher M.P., Magruder K. (2017). Military service member and veteran reintegration: A critical review and adapted ecological model. Am. J. Orthopsychiatry.

[8] Cheney A.M., Koenig C.J., Miller C.J., Zamora K., Wright P., Stanley R., Fortney J., Burgess J.F., Pyne J.M. (2018). Veteran-centered barriers to VA mental healthcare services use. BMC Health Serv. Res..

[9] Vogt D. (2011). Mental health-related beliefs as a barrier to service use for military personnel and veterans: A review. Psychiatr. Serv..

[10] Kondo K., Low A., Everson T., Gordon C.D., Veazie S., Lozier C.C., Freeman M., Motúapuaka M., Mendelson A., Friesen M. (2017). Health disparities in veterans: A map of the evidence. Med. Care.

[11] United States-México Border Health Commission (2014). Access to Health Care in the U.S.-México Border Region: Challenges and Opportunities. https://www.ruralhealthinfo.org/assets/939-3103/access-to-health-care-u.s.-mexico-border.pdf.

[12] Duenas K.R., Ingram M., Crocker R.M., Pace T.W.W., de Zapien J.G., Torres E., Carvajal S.C. (2022). La Vida en la Frontera: Protocol for a prospective study exploring stress and health resiliencies among Mexican-origin individuals living in a U.S.-Mexico border community. BMC Public Health.

[13] Pew Research Center (2024). How Americans View the Situation at the U.S.-Mexico Border, Its Causes and Consequences. https://www.pewresearch.org/politics/2024/02/15/how-americans-view-the-u-s-mexico-border-situation-and-the-governments-handling-of-the-issue/.

[14] Carvajal S.C., Rosales C., Rubio-Goldsmith R., Sabo S., Ingram M., McClelland D.J., Redondo F., Torres E., Romero A.J., O’Leary A.O. (2013). The Border Community and Immigration Stress Scale: A preliminary examination of a community-responsive measure in two southwest samples. J. Immigr. Minor. Health.

[15] O’Connor K., Anders R.L., Balcazar H., Ibarra J., Perez E., Flores L., Ortiz M., Bean N.H. (2008). Prevalence of mental health issues in the borderlands: A comparative perspective. Hisp. Health Care Int..

[16] Merians A.N., Gross G., Spoont M.R., Bellamy C.D., Harpaz-Rotem I., Pietrzak R.H. (2023). Racial and ethnic mental health disparities in U.S. military veterans: Results from the National Health and Resilience in Veterans Study. J. Psychiatr. Res..

[17] Ward R.E., Nguyen X.T., Li Y., Lord E.M., Lecky V., Song R.J., Casas J.P., Cho K., Gaziano J.M., Harrington K.M. (2021). Racial and ethnic disparities in U.S. veteran health characteristics. Int. J. Environ. Res. Public Health.

[18] Centers for Disease Control and Prevention (2024). Fast Facts: Health and Economic Costs of Chronic Conditions. https://www.cdc.gov/chronic-disease/data-research/facts-stats/index.html.

[19] Pillai D., Artiga S. (2022). Health and Health Care in the U.S.-Mexico Border Region. https://www.kff.org/racial-equity-and-health-policy/issue-brief/health-and-health-care-in-the-u-s-mexico-border-region/.

[20] Ravindran C., Morley S.W., Stephens B.M., Stanley I.H., Reger M.A. (2020). Association of suicide risk with transition to civilian life among US military service members. JAMA Netw. Open.

[21] Sayer N.A., Noorbaloochi S., Frazier P., Carlson K., Gravely A., Murdoch M. (2010). Reintegration problems and treatment interests among Iraq and Afghanistan combat veterans receiving VA medical care. Psychiatr. Serv..

[22] Betancourt J.A., Granados P.S., Pacheco G.J., Reagan J., Shanmugam R., Topinka J.B., Beauvais B.M., Ramamonjiarivelo Z.H., Fulton L.V. (2021). Exploring health outcomes for U.S. veterans compared to non-veterans from 2003 to 2019. Healthcare.

[23] Military Health System (2023). Combat and Operational Stress Reactions (COSRs). https://www.health.mil/Military-Health-Topics/Centers-of-Excellence/Psychological-Health-Center-of-Excellence/Psychological-Health-Readiness/Combat-and-Operational-Stress-Control/COSRs.

[24] Kaplan M.S., McFarland B.H., Huguet N., Valenstein M. (2012). Suicide risk and precipitating circumstances among young, middle-aged, and older male veterans. Am. J. Public Health.

[25] Blais R.K., Tsai J., Southwick S.M., Pietrzak R.H. (2015). Barriers and facilitators related to mental health care use among older veterans in the United States. Psychiatr. Serv..

[26] Maguen S., Madden E., Cohen B.E., Bertenthal D., Neylan T.C., Seal K.H. (2015). Suicide risk in Iraq and Afghanistan veterans with mental health problems in VA care. J. Psychiatr. Res..

[27] Washington D.L., Yuan A., Toyama J.A., Jackson L., Kasom D.R., Canning M., Steers W.N. (2022). National Veteran Health Equity Report 2021. Focus on Veterans Health Administration Patient Experience and Health Care Quality. https://www.va.gov/HEALTHEQUITY/docs/NVHER_2021_Report_508_Conformant.pdf.

[28] Lehavot K., Hoerster K.D., Nelson K.M., Jakupcak M., Simpson T.L. (2012). Health indicators for military, veteran, and civilian women. Am. J. Prev. Med..

[29] Teeters J.B., Lancaster C.L., Brown D.G., Back S.E. (2017). Substance use disorders in military veterans: Prevalence and treatment challenges. Subst. Abuse Rehabil..

[30] Olenick M., Flowers M., Diaz V.J. (2015). US veterans and their unique issues: Enhancing health care professional awareness. Adv. Med. Educ. Pract..

[31] Meisler A.W., Gianoli M.O., Na P.J., Pietrzak R.H. (2023). Functional disability in US military veterans: The importance of integrated whole health initiatives. Prim. Care Companion CNS Disord..

[32] Stika M.M., Riordan P., Aaronson A., Herrold A.A., Ellison R.L., Kletzel S., Drzewiecki M., Evans C.T., Mallinson T., High W.M. (2021). Cognition and other predictors of functional disability among veterans with mild traumatic brain injury and posttraumatic stress disorder. J. Head Trauma Rehabil..

[33] Clement S., Williams P., Farrelly S., Hatch S.L., Schauman O., Jeffery D., Henderson R.C., Thornicroft G. (2015). Mental health-related discrimination as a predictor of low engagement with mental health services. Psychiatr. Serv..

[34] Possemato K., Wray L.O., Johnson E., Webster B., Beehler G.P. (2018). Facilitators and barriers to seeking mental health care among primary care veterans with posttraumatic stress disorder. J. Trauma. Stress.

[35] Sharifian N., Kolaja C.A., LeardMann C.A., Castañeda S.F., Carey F.R., Seay J.S., Carlton K.N., Rull R.P., Cohort Study Team F.T.M. (2024). Racial, ethnic, and sex disparities in mental health among US service members and veterans: Findings from the Millennium Cohort Study. Am. J. Epidemiol..

[36] Washington D.L., Bean-Mayberry B., Riopelle D., Yano E.M. (2011). Access to care for women veterans: Delayed healthcare and unmet need. J. Gen. Intern. Med..

[37] MacDonald S., Judge-Golden C., Borrero S., Zhao X., Mor M.K., Hausmann L.R.M. (2020). Experiences of perceived gender-based discrimination among women veterans: Data from the ECUUN Study. Med. Care.

[38] Galovski T.E., Street A.E., Creech S., Lehavot K., Kelly U.A., Yano E.M. (2022). State of the knowledge of VA military sexual trauma research. J. Gen. Intern. Med..

[39] Denise E.J. (2014). Multiple disadvantaged statuses and health. J. Health Soc. Behav..

[40] Harnois C.E. (2014). Are perceptions of discrimination unidimensional, oppositional, or intersectional? Examining the relationship among perceived racial-ethnic-, gender-, and age-based discrimination. Sociol. Perspect..

[41] Lehavot K., Katon J.G., Chen J.A., Fortney J.C., Simpson T.L. (2018). Post-traumatic stress disorder by gender and veteran status. Am. J. Prev. Med..

[42] Fortuna L.R., Alegria M., Gao S. (2010). Retention in depression treatment among ethnic and racial minority groups in the United States. Depress. Anxiety.

[43] Bennett E., Crabtree M., Schaffer M., Britt T. (2011). Mental health status and perceived barriers to seeking treatment in rural reserve component veterans. J. Rural Soc. Sci..

[44] Demers A.L. (2013). From death to life. J. Humanist. Psychol..

[45] Trautmann J., Alhusen J., Gross D. (2015). Impact of deployment on military families with young children: A systematic review. Nurs. Outlook.

[46] Leslie L.A., Koblinsky S.A. (2017). Returning to civilian life: Family reintegration challenges and resilience of women veterans of the Iraq and Afghanistan wars. J. Fam. Soc. Work.

[47] Knobloch L.K., Theiss J.A. (2011). Depressive symptoms and mechanisms of relational turbulence as predictors of relationship satisfaction among returning service members. J. Fam. Psychol..

[48] Campbell S.B., Renshaw K.D. (2018). Posttraumatic stress disorder and relationship functioning: A comprehensive review and organizational framework. Clin. Psychol. Rev..

[49] Mankowski M., Haskell S.G., Brandt C., Mattocks K.M. (2015). Social support throughout the deployment cycle for women veterans returning from Iraq and Afghanistan. Soc. Work Health Care.

[50] Morin R. (2011). The Difficult Transition from Military to Civilian Life. https://www.pewresearch.org/social-trends/2011/12/08/the-difficult-transition-from-military-to-civilian-life/.

[51] Koenig C.J., Maguen S., Monroy J.D., Mayott L., Seal K.H. (2014). Facilitating culture-centered communication between health care providers and veterans transitioning from military deployment to civilian life. Patient Educ. Couns..

[52] Wilson G., Hill M., Kiernan M.D. (2018). Loneliness and social isolation of military veterans: Systematic narrative review. Occup. Med..

[53] Haynie J.M., Shepherd D. (2011). Toward a theory of discontinuous career transition: Investigating career transitions necessitated by traumatic life events. J. Appl. Psychol..

[54] Randles R., Finnegan A. (2022). Veteran help-seeking behaviour for mental health issues: A systematic review. BMJ Mil. Health.

[55] Abdisa E., Fekadu G., Girma S., Shibiru T., Tilahun T., Mohamed H., Wakgari A., Takele A., Abebe M., Tsegaye R. (2020). Self-stigma and medication adherence among patients with mental illness treated at Jimma University Medical Center, Southwest Ethiopia. Int. J. Ment. Health Syst..

[56] Eylem O., de Wit L., van Straten A., Steubl L., Melissourgaki Z., Danışman G.T., de Vries R., Kerkhof A.J.F.M., Bhui K., Cuijpers P. (2020). Stigma for common mental disorders in racial minorities and majorities a systematic review and meta-analysis. BMC Public Health.

[57] U.S. Department of Health and Human Services Office of Minority Health (2024). Mental and Behavioral Health—Hispanics. https://minorityhealth.hhs.gov/mental-and-behavioral-health-hispanics#.

[58] Oh H., Trinh M.P., Vang C., Becerra D. (2024). Addressing barriers to primary care access for Latinos in the U.S.: An agent-based model. Soc. Serv. Rev..

[59] Forcén F.E., Vélez Flórez M.C., Bido Medina R., Zambrano J., Pérez J.H., Rodríguez A.M., Santos L.H. (2023). Deconstructing cultural aspects of mental health care in Hispanic/Latinx people. Psychiatr. Ann..

[60] Hennink M., Kaiser B.N. (2022). Sample sizes for saturation in qualitative research: A systematic review of empirical tests. Soc. Sci. Med..

[61] DeJonckheere M., Vaughn L.M. (2019). Semistructured interviewing in primary care research: A balance of relationship and rigour. Fam. Med. Commun. Health.

[62] Naeem M., Ozuem W., Howell K., Ranfagni S. (2023). A step-by-step process of thematic analysis to develop a conceptual model in qualitative research. Int. J. Qual. Methods.

[63] Braun V., Clarke V. (2006). Using thematic analysis in psychology. Qual. Res. Psychol..

[64] Nowell L.S., Norris J.M., White D.E., Moules N.J. (2017). Thematic analysis: Striving to meet the trustworthiness criteria. Int. J. Qual. Methods.

[65] U.S. Department of Veterans Affairs (2015). What It Means to Be Mission Oriented. https://www.va.gov/vetsinworkplace/docs/em_missionOriented.asp.

[66] Gilligan C. (2022). Who Are America’s Veterans?. https://www.usnews.com/news/best-states/articles/2022-11-11/who-are-americas-veterans.

[67] Watson Institute for International and Public Affairs (2021). U.S. Veterans & Military Families. https://watson.brown.edu/costsofwar/costs/human/veterans.

[68] U.S. Department of Veterans Affairs VA Research on Mental Health. https://www.research.va.gov/topics/mental_health.cfm.

[69] U.S. Government Accountability Office (2021). Veterans’ Growing Demand for Mental Health Services. https://www.gao.gov/products/gao-21-545sp.

[70] Pew Research Center (2011). War and Sacrifice in the Post-9/11 Era. https://www.pewresearch.org/social-trends/2011/10/05/war-and-sacrifice-in-the-post-911-era/.

[71] Tanielian T., Woldetsadik M.A., Jaycox L.H., Batka C., Moen S., Farmer C., Engel C.C. (2016). Barriers to engaging service members in mental health care within the U.S. military health system. Psychiatr. Serv..

[72] Blosnich J.R., Dichter M.E., Cerulli C., Batten S.V., Bossarte R.M. (2014). Disparities in adverse childhood experiences among individuals with a history of military service. JAMA Psychiatry.

[73] Kulesza M., Pedersen E., Corrigan P., Marshall G. (2015). Help-seeking stigma and mental health treatment seeking among young adult veterans. Mil. Behav. Health.

[74] Goldberg S.B., Simpson T.L., Lehavot K., Katon J.G., Chen J.A., Glass J.E., Schnurr P.P., Sayer N.A., Fortney J.C. (2019). Mental health treatment delay: A comparison among civilians and veterans of different service eras. Psychiatr. Serv..

[75] Johnson T.S., Ganz A., Berger S., Ganguly A., Koritzky G. (2018). Service members prefer a psychotherapist who is a veteran. Front. Psychol..

[76] Centers for Disease Prevention and Control (2023). Health Disparities. https://www.cdc.gov/healthyyouth/disparities/index.htm.

[77] Cook B.L., Hou S.S., Lee-Tauler S.Y., Progovac A.M., Samson F., Sanchez M.J. (2019). A Review of mental health and mental health care disparities research: 2011–2014. Med. Care Res. Rev..

[78] Maura J., Weisman de Mamani A. (2017). Mental health disparities, treatment engagement, and attrition among racial/ethnic minorities with severe mental illness: A Review. J. Clin. Psychol. Med. Settings.

[79] McClendon J., Perkins D., Copeland L.A., Finley E.P., Vogt D. (2019). Patterns and correlates of racial/ethnic disparities in posttraumatic stress disorder screening among recently separated veterans. J. Anxiety Disord..

[80] Maguen S., Ren L., Bosch J.O., Marmar C.R., Seal K.H. (2010). Gender differences in mental health diagnoses among Iraq and Afghanistan veterans enrolled in Veterans Affairs Health Care. Am. J. Public Health.

[81] Smith D.T., Mouzon D.M., Elliott M. (2018). Reviewing the assumptions about men’s mental health: An exploration of the gender binary. Am. J. Men’s Health.

[82] Seal K.H., Maguen S., Cohen B., Gima K.S., Metzler T.J., Ren L., Bertenthal D., Marmar C.R. (2010). VA mental health services utilization in Iraq and Afghanistan veterans in the first year of receiving new mental health diagnoses. J. Trauma. Stress.

[83] U.S. Department of Health and Human Services Mental Health and Mental Disorders Evidence-Based Resources. https://health.gov/healthypeople/objectives-and-data/browse-objectives/mental-health-and-mental-disorders/evidence-based-resources.

[84] Kleykamp M., Montgomery S., Pang A., Schrader K. (2021). Military identity and planning for the transition out of the military. Mil. Psychol..

[85] Maguen S., Lucenko B.A., Reger M.A., Gahm G.A., Litz B.T., Seal K.H., Knight S.J., Marmar C.R. (2010). The impact of reported direct and indirect killing on mental health symptoms in Iraq war veterans. J. Trauma. Stress.

[86] Currier J.M., Holland J.M., Malott J. (2015). Moral injury, meaning making, and mental health in returning veterans. J. Clin. Psychol..

[87] Eghaneyan B.H., Murphy E.R. (2020). Measuring mental illness stigma among Hispanics: A systematic review. Stigma Health.

[88] Knowles K.A., Sripada R.K., Defever M., Rauch S.A.M. (2019). Comorbid mood and anxiety disorders and severity of posttraumatic stress disorder symptoms in treatment-seeking veterans. Psychol. Trauma.

[89] Mulvaney-Day N., Marshall T., Downey Piscopo K., Korsen N., Lynch S., Karnell L.H., Moran G.E., Daniels A.S., Ghose S.S. (2018). Screening for behavioral health conditions in primary care Settings: A systematic review of the literature. J. Gen. Intern. Med..

[90] American Psychiatric Association (2017). Mental Health Disparities: Hispanics and Latinos. https://www.psychiatry.org/File%20Library/Psychiatrists/Cultural-Competency/Mental-Health-Disparities/Mental-Health-Facts-for-Hispanic-Latino.pdf.

