# Supplementary material for: Mental Health Challenges and Barriers to Veterans’ Adjustment to Civilian Life on the U.S.–Mexico Border
Source: Healthcare (Basel). 2025 Jan 22;13(3):220. doi: 10.3390/healthcare13030220 (PMC11817780; doi:10.3390/healthcare13030220)
Supplement: Supplementary file 1 [file healthcare-13-00220-s001.zip › healthcare-3375031-supplementary.pdf]

Supplementary Materials

Interview Questions

Demographics

1. How old are you?
2. How far is your education?
3. Are you currently married?
4. Do you have any children? What are their ages?
5. What is your ethnic/racial classification?
6. Were you born in the U.S.? If not, what is your birth country?
7. How long have you resided in El Paso?

Services at [Name of Agency]

8. What services are you currently receiving at the [Name of Agency]?
9. What are the reasons that you are seeking services at [Name of Agency]?
10. How long have you received mental health treatment from [Name of Agency]?

Mental health

11. Can you describe some of the mental health conditions that you are currently experiencing?
12. How long have you felt this way?
13. Do you have any mental health issues in your childhood? If so, can you describe your experience?
14. Have you ever received mental health services elsewhere?
15. Do you have any other mental health diagnoses?
16. How has your current mental health status affected your daily life?

Social relationships

17. Does any of your family members have a history of mental illness?
18. Can you describe your relationship with your family of origin?
19. Whom do you currently live with?
20. Do you feel like your mental health condition has had any impact on your relationship with your family?
21. Has the nature of the relationship changed for you before and after receiving mental health treatment?

Employment

22. Are you currently employed? If so, what do you do for a living?

23. How long have you been working at your current employment?
24. Can you describe your employment history?
25. Do you feel that your mental health condition has any impact on your employment?
26. Has the relationship with your employment for you changed before and after receiving mental health treatment?

Education

27. Are you currently in school?
28. Do you feel that your mental health condition has any impact on your education?
29. Has the relationship with your schooling for you changed before and after receiving mental health treatment?

Outlook of life

30. Can you describe some factors related to your mental health that have limited your life opportunities?
31. Can you describe your plan for the future?
32. Do you think you have control over your life?

Support and assistance

33. Do you currently receive any social support? If so, can you describe the type of support you received?
34. Do you currently receive any financial assistance? If so, from whom do you receive such support?
35. Do you have other economic means to support yourself (besides employment)?
36. Have you ever used other community services to cope with mental health?

Personal histories

37. Have you ever been arrested or become incarcerated? If so, can you briefly describe the reasons for your arrest or the type of your offense?
38. Do you currently have any other undiagnosed mental illness?
39. Do you currently have any health issues?
40. Have you ever become/felt victimized in your life?

Veteran

41. How long ago did you serve in the military?
42. How long have you served?
43. What did you do in the military?
44. How are you adjusting to life as military personnel versus as a civilian?

45. Do you feel that serving in the military has any impact on your mental health?
46. Do you maintain any relationships with other veterans coping with mental health issues? If so, can you describe it?
47. Can you describe public perception, reaction, or treatment toward veterans?
48. Have you ever been stigmatized as a veteran? Can you describe the circumstance?
49. Can you describe your impression about the public policies you know of that are related to treatment toward the military?

Mental health treatment

50. Are you currently taking any medication prescribed for your mental health?
51. Are you taking the medication as prescribed?
52. Are you attending your mental health appointments regularly?
    a. Are you able to regularly keep your appointments with [Name of Agency]?
    b. If not, what tends to keep you from attending regularly?
    c. Do transportation issues make it difficult to keep your [Name of Agency] appointments regularly?
    d. Do work schedules make it difficult to keep your [Name of Agency] appointments regularly?
    e. Do school schedules make it difficult to keep your [Name of Agency] appointments regularly?
    f. Do child or other family care issues make it difficult to keep your [Name of Agency] appointments regularly?
    g. Could [Name of Agency] offer appointments at another time that would make it easier for you to keep your [Name of Agency] appointments regularly?
    h. Does anxiety or fear of discussing sensitive information make it difficult to keep your [Name of Agency] appointments regularly?
    i. Do issues with controlled substances or alcohol make it difficult to keep your [Name of Agency] appointments regularly?
    j. Does depression make it difficult to keep your [Name of Agency] appointments regularly?
    k. Does fatigue make it difficult to keep your [Name of Agency] appointments regularly?
    l. Can we have your permission to review your appointment log at [Name of Agency]?

Barriers

53. What are the challenges that you are currently facing as a veteran experiencing mental health needs?
54. What barriers are you currently facing in improving your mental health?
55. What are the strategies that you have used to improve your mental health?
56. Can you describe your satisfaction or dissatisfaction with the mental health services that you are receiving at [Name of Agency]?
57. Do you have any suggestions you can share to improve the services that [Name of Agency] can provide you?

Survey Questions

1. Strongly disagree
2. Disagree
3. Neither disagree nor agree
4. Agree
5. Strong agree

1. I feel hopeless about my current living situation.
2. I am depressed about my current living situation.
3. I feel frustrated about my current living situation.
4. I feel lost about my current living situation.
5. I feel vulnerable about my current living situation.
6. I worried a lot about my current living situation.
7. In general, I am happy about my current living situation.
8. I have good coping skills.
9. I have good interpersonal and communication skills.
10. I have competent life skills to survive on my own.
11. I have good money management skills.
12. I have substance abuse issues that have hindered my ability to function daily.
13. I have mental health issues that have hindered my ability to function daily.
14. I have health issues that have hindered my ability to function daily.
15. I have financial challenges that have hindered my ability to function daily.
16. I have trouble getting a job.
17. I have trouble keeping a job.
18. I can plan for my future on my own.
19. I have a strong will to overcome my mental health challenges.
20. I believe in myself and my ability to succeed.
21. I feel worthless.
22. My current mental health challenges are only temporary.
23. I have someone to turn to if I need help.
24. I am familiar with the resources available for the veteran population in El Paso.
